# Routinely Performed Serial Follow-Up Imaging in Asymptomatic Patients With Multiple Cerebral Cavernous Malformations Has No Influence on Surgical Decision Making

**DOI:** 10.3389/fneur.2018.00848

**Published:** 2018-10-11

**Authors:** Julia Velz, Martin Nikolaus Stienen, Marian Christoph Neidert, Yang Yang, Luca Regli, Oliver Bozinov

**Affiliations:** ^1^Department of Neurosurgery, Clinical Neuroscience Center, University Hospital Zurich, Zurich, Switzerland; ^2^University of Zurich, Zurich, Switzerland

**Keywords:** cerebral cavernous malformation, CM, mCCM, cavernoma, cavernous angioma, cavernous hemangioma, follow-up

## Abstract

**Background:** The best strategy to perform follow-up of patients with multiple cerebral cavernous malformations (mCCM) is unclear due to the unpredictable clinical course. Still, serial radiological follow-up is often performed. The objective of this work was to critically question whether active follow-up by serial imaging is justified and has an impact on clinical decision making.

**Methods:** We included all consecutive patients with mCCM treated and followed at our Department between 2006 and 2016. Patient data were collected and analyzed retrospectively.

**Results:** From a total number of 406 patients with CCM, *n* = 73 [18.0%; mean age at first diagnosis 45.2 years (±2.4 SE); *n* = 42 male (57.5 %)] were found to harbor multiple lesions (≤5 CCM in 58.9%; 6–25 in 21.9%; ≥ 25 in 19.2%). All of them were followed for a mean of 6.8 years (±0.85 SE). Conservative treatment was suggested in 43 patients over the complete follow-up period. Thirty patients underwent surgical extirpation of at least one CCM lesion. Forty-three surgical procedures were performed in total. During 500.5 follow-up years in total, routinely performed follow-up MRI in asymptomatic patients lead to an indication for surgery in only two occasions and even those two were questionable surgical indications.

**Conclusion:** Routinely performed follow-up MRI in asymptomatic patients with mCCM is highly questionable as there is no evidence for therapeutic relevance.

## Introduction

CCM are the most common vascular malformations in the brain with an incidence of ~0.4–0.6% (1, 2). The clinical presentation of patients with CCM ranges from incidental findings in MRI to symptomatic courses with focal neurologic deficits and epilepsy (2). While patients with single lesions often have a sporadic form of the disease, the number of patients harboring multifocal lesions, associated with familial CCM (FCCM), is high (10–31% of all cases). The principle danger inherent to CCM is the hemorrhage risk that has been estimated around 0.6–2% per lesion-year or 4.3–13% per patient-year in prospective studies with FCCM (3–5). While no definite evidence is suggested to correlate between hemorrhagic risk and size, age, sex, multiplicity or associated DVA, prior hemorrhage and brainstem CCM location were found to be the only significant risk factors for CCM hemorrhage (6, 7). Whereas in other vascular malformations (i.e., aneurysms, arteriovenous malformations) grading systems have been established to estimate the risk of intracerebral hemorrhage (ICH), so far no grading system exists in the literature to serve as a valuable aid in the clinical decision as to whether perform conservative or surgical treatment in patients with CCM (8–10).

To date, irrespective of location, asymptomatic CCM are mainly observed (Class III, Level B) (11, 12). Microsurgical resection might be considered in patients with asymptomatic CCM in non-eloquent areas to prevent future hemorrhage when lifestyle, occupation and the psychological burden outweigh the risk of surgical morbidity (Class IIb, Level C) (12). For patients with incidental diagnosis of mCCM, microsurgical resection is not recommended, given the risk, as well as the morbidity associated with the multiple surgical approaches (Class III, Level B) (11). Surgical resection is recommended in patients with symptomatic CCM located in non-eloquent areas, which present with new focal neurologic deficit (Class IIb, Level B), recurrent hemorrhage and medical refractory epilepsy (Class IIa, Level B) (12). In these cases, surgery is generally safe and can prevent re-bleeding on one hand and show efficacy for control of epilepsy on the other hand (13–16).

As the clinical course of patients with mCCM is unpredictable, there is controversery whether or not active, serial clinical and radiological follow-up should be recommended (11, 12, 17). As little data is available on this issue, indication and timing of follow-up imaging in patients with CCM as well as mCMM is based on individual judgment, insurance and patient preferences for now (12). Since no data exists in the literature on follow-up strategies of patient with mCCM, the objective of this study was to critically question whether active follow-up by serial imaging has an impact on clinical decision making in patients with mCCM and can prevent CCM hemorrhage in the further course. Our data of our patient cohort with single CCM will be reported separately.

## Materials and methods

### Patient population

Retrospective single-center study, encompassing all patients with CCM that presented at the Department of Neurosurgery at the University Hospital Zurich between 2006 and 2016. Patients fulfilling the following criteria were eligible and included: (1) radiological or histological diagnosis of ≥ 2 CCM (= mCCM) (2) follow-up data were available. Radiological and histological diagnosis of mCCM were confirmed either by a senior neuroradiologist and/or neuropathologist.

### Data collection

The patient's medical history was analyzed for age, gender and clinical symptoms (focal neurologic deficit and/or epilepsy). Headache was not considered as a clinical symptom of CCM bleeding in this study. In addition, date of first diagnosis, date of first contact to a neurosurgical department, date of last follow-up, as well as the number and dates of MRI studies were collected. MRI studies always comprised at least T1-weighted imaging (with and without contrast enhancement), T2-weighted imaging, and gradient echo sequences in all three planes (axial, coronal, and sagittal) or susceptibility-weighted imaging (SWI). MRI-studies were analyzed in regards of CCM location (supratentorial, infratentorial, spinal) and CCM number, considering the natural course with decrease or increase in size and *de novo*-synthesis. A CCM-related hemorrhage was defined with radiological evidence (CT or MRI) of subacute or acute hemorrhage compared to previous imaging. In patients with initial ICH suspicious of CCM hemorrhage, the diagnosis of CCM was either confirmed by imaging during follow-up or after CCM resection in the further course. The dates and indications for each surgical intervention were obtained, as was the type of patient contact—being either elective (out-patient consultation) or emergency (in-patient consultation/emergency room).

### Patient management

At our department, we usually do not operate on asymptomatic patients with incidental diagnosis of CCM. We consider complete surgical resection in symptomatic patients with first-ever hemorrhagic or epileptic presentation if the CCM is localized non-eloquent and can be removed with a small risk for postoperative morbidity. In symptomatic patients with deep-seated or (very) eloquent CCM (e.g., brain stem, basal ganglia) a more conservative attitude is taken, and we restrain from operating after the first hemorrhage or when little/spontaneously improving neurological deficits occur. In our department microsurgical resection is the preferred treatment option and stereotactic radiosurgery is not applied in patients with CCM. We do perform routine radiological and clinical follow-up yearly in patients with mCCM, comprising both MRI studies and consultation with a neurovascular surgeon. Physical as well as neurological examination are performed to determine whether a newly discovered CCM or increase in size of a CCM is truly asymptomatic or whether a patient has experienced a history of seizures or an undiagnosed neurological deficit.

### Ethical considerations

The study was approved by the Cantonal Ethics Committee (KEK-ZH; application number 2017-00330).

### Statistical analysis

The software used for descriptive statistical analysis was Stata v14.2 for Mac (StataCorp, College Station, Texas, USA).

## Results

**Figure 1:**
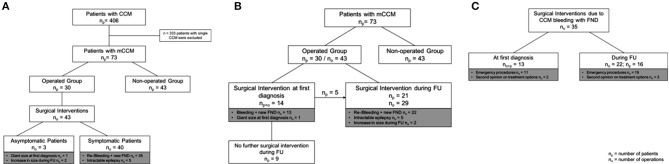
**(A)** Analyzed study cohort: From 406 patients with CCM, 73 (18.0%) patients harbored mCCM; *n* = 30 were operated and *n* = 43 patients were not operated. Forty-three surgical interventions were performed in total. Forty surgical interventions were performed in symptomatic patients and 3 surgical interventions were performed in asymptomatic patients. **(B)** Timepoint of surgical intervention: 14 surgical interventions were performed at first diagnosis and 29 surgical interventions were performed during FU. **(C)** Surgical Interventions due to CCM bleeding with FND: In 13 patients and 13 instances surgical interventions were performed at first diagnosis; whereas in 16 patients and 22 instances surgical intervention were performed during FU; CCM, cerebral cavernous malformations; n_p_, number of patients; n_o_, number of operations; FU, follow-up; FND, focal neurological deficits.

Four hundred and six patients with CCM were seen at our institution and 73 (18.0%) patients harbored mCCM, fulfilled the above criteria for this study, and were included in the analysis (Figure 1). Follow-up data were available for all 73 patients with mCCM.

### Patient population

The mean age at first diagnosis was 45.2 years (± 2.4 SE); 57.5% of the patients were male (Table 1). CCM were present in the supratentorial (90.4%), infratentorial (68.5%) and spinal compartments (2.7%). Forty-three patients (58.9%) harbored ≤ 5 CCM; whereas in 16 patients (21.9%) 6-25 CCM and in 14 patients (19.2%) ≥25 CCM were found. Twenty-seven patients (37.0%) stayed free of symptoms during follow-up, 46 patients (63.0%) presented with either seizures (*n* = 21, 28.8%) and/or neurological deficits (*n* = 34, 46.6%). The mean follow-up was 6.8 (± 0.85 SE) years per patient (range 0.12–35.0 yrs). The total follow-up time was 500.5 person-years.

**Table 1:** Baseline patient characteristics.

Age in years (mean ± SE)	45.2	±2.4
Sex		
Male Female	42 31	57.5% 42.5%
Location^*^		
Supratentorial Infratentorial Spinal	66 50 2	90.4% 68.5% 2.7%
Number of CCM		
≤ 5 6–25 ≥ 25	43 16 14	58.9% 21.9% 19.2%
Symptomatic - Epilepsy - Neurological deficit Asymptomatic	46 21 34 27	63.0% 28.8% 46.6% 37.0%
Follow-up time in years (mean ± SE)	6.8	0.85

*CCM, cerebral cavernous malformations; SE, standard error*.

* *Does not add up to 100% as most patients with mCCM presented lesions in multiple compartments*.

### Clinical management—conservative and operative treatment

Conservative treatment was performed in 43 patients, while 30 patients underwent surgical extirpation of at least one CCM lesion (Figure 1). The length of follow-up was 10.3 (±1.6 SE) in the operated and 4.5 (±0.7 SE) years per patient in the non-operated patient group. Among the 30 patients that underwent surgery, 43 surgical procedures were performed in total. Amongst the 43 surgical interventions that were performed, in 40 cases the patient was symptomatic and presented with either new focal neurological deficits due to CCM (re-)bleeding (*n* = 35) or intractable epilepsy (*n* = 5). In only three instances surgery was performed in patients who were asymptomatic regarding the CCM that was resected; in these cases, surgery was performed due to a giant size of the lesion at first diagnosis (*n* = 1) or increase in size during follow-up (*n* = 2).

At time of first patient contact surgical resection of CCM was performed in 14 patients and 14 instances. During follow-up in 21 patients and in 29 instances surgical treatment was performed. Among these 21 patients, that underwent treatment during follow-up 38.1% (*n* = 8) had already undergone CCM resection at least once. In two patients, CCM extirpation and hematoma evacuation was performed four and five times, respectively, due to recurrent CCM bleeding with acute neurological deficits.

### Surgical intervention due to new focal neurological deficits

Among the 35 surgical interventions that were performed due to CCM bleeding with new focal neurological deficits, in 30 instances (85.7%) patients presented to the emergency unit with acute CCM bleeding and severe neurological deficits, whereas in five instances (14.3%) patients were referred to our department for a second opinion on surgical treatment options [*n* = 4 due to (re-)bleeding of pontine CCM and *n* = 1 due to bleeding of a CM in the spinal cord].

Amongst the 35 surgical interventions that were performed due to CCM bleeding with new focal neurological deficits, in 13 patients and 13 instances surgical treatment was performed after first diagnosis of CCM, whereas in 16 patients and 22 instances surgical intervention was performed during follow-up. Among the 22 instances where surgery was performed during follow-up, in 19 instances (86.4%) the patient presented themselves at the emergency room and in three instances (13.6%) the patients were referred to our department for second-opinion on surgical treatment options [*n* = 3 due to (re-)bleeding of pontine CCM].

### Surgical intervention due to intractable epilepsy

Considering the five instances, where surgery was performed due to intractable epilepsy, all patients (*n* = 4) were seen during consultation and decision for surgery was done during follow-up. In one patient, surgery was performed twice due to intractable epilepsy. None of the patients stayed seizure free after surgery.

### Surgical intervention in asymptomatic patients

Three patients were operated on asymptomatic lesions. In those, indication for surgery was done after first diagnosis in one patient (Case 1) and during follow-up in two patients (Case 2/3): Case 1: Surgery was performed in a young child, who showed among mCCM one fronto-basal CCM measuring 6 × 3 cm with close proximity to basal ganglia. Case 2: Surgery was performed in a young adult, who showed an increase in size of an asymptomatic CCM lesion in the left frontomedial gyrus measuring 2.3 × 2.3 cm during follow-up. Case 3: Surgery was performed in a young adult with residual right-sided hemiparesis due to bleeding of a left-sided ventral pontine CCM (and resection 2 years previously), who showed during follow-up an increase in size of a CCM in right anterior putamen.

### Impact of active follow-up on surgical decision making

In summary, during 500.5 follow-up years in total in only 2/73 patients with mCCM (2.7%) surgical intervention was performed due to proactive follow-up by MRI, although patients were asymptomatic, regarding the CCM lesion that was resected.

Among the 35 instances where surgery was performed due to CCM (re-)bleeding with new focal neurological deficits in 30 instances (85.7%) patients presented to the emergency unit. During follow-up, the percentage of patients presenting at the emergency unit due to CCM bleeding with new focal neurological deficits stayed high with 86.4%; in three instances (13.6%) the patients were referred to our department for second-opinion on surgical treatment options during follow-up. No patient with CCM (re-)bleeding and new focal neurological deficit was seen during routinely performed follow-up.

## Discussion

The course of patients with mCCM is heterogeneous, as some present with stable lesions for many years, while in others lesion size and number increases or less often decreases over time. To date, there is no consensus on the best management for mCCM either by active follow-up and serial imaging, or by specific imaging only in case of new onset of clinical symptoms (uncommon headache, neurological deficit, or seizure) (12, 17, 18). As little data is available on this issue, indication and timing of follow-up imaging is based on individual judgment, insurance and patient preferences for now, and relatively little evidence is available to make recommendations (12, 17, 18).

### Follow-up strategy

For patients with single but also multiple CCM we suggested previously to perform an initial MRI postoperatively or days after the first hemorrhage, and a follow-up MRI 2–3 months afterwards. Yearly MRI were considered for all patients with or without surgery and should be performed in a neurosurgical experienced center (5). This recommendation was based on multiple earlier recommendations (12, 17). Considering the present data, however, we now focus on thorough patient education about their disease and its natural course. Given the fact that the risk for a detrimental hemorrhage is generally very low (3–5), we suggest no active follow-up in asymptomatic patients with mCCM who feel comfortable about this. Patients receive detailed contact information for our emergency department and can contact us 24/7 in case of new-onset of symptoms. For patients that are more concerned and request a more proactive follow-up, we offer this according to our previous follow-up plan, outlined above.

In patients with asymptomatic but growing lesions, one might feel less confident about not proactively following those patients by serial imaging. It must be emphasized, however, that increase in lesion size does not indicate higher risk of hemorrhage, and the decision to operate is only rarely based on lesion growth in otherwise oligo- or asymptomatic patients (19). In this particular situation, the follow-up plan should be decided individually on a case-to-case basis, but we also consider abstaining from serial imaging an adequate option here.

Follow-up should be performed in a neuroscience experienced center—neurologists as well as neuroradiologists can follow stable patients. Patients with symptomatic epilepsy should be supervised by neurologists. Patient referral to a neurosurgeon should be considered for evaluation of symptomatic patients or progressive lesions, as the only treatment option proven to be effective is surgical.

Whereas the percentage of patients presenting with ICH due to CCM hemorrhage at first diagnosis is hard to minimize, the aim of routinely performed follow-up MRI in patients which harbor mCCM is to prevent CCM hemorrhage in the further course by early surgical recommendation based on patients and radiographic factors. However, a model based on patients and radiographic factors, which predicts the lifetime rupture risk of CCM in each individual case in order to decide whether surgical treatment should be recommended or not is missing in the literature—consecutively the therapeutic relevance of routinely performed follow-up MRI is very limited.

In this study, our patient cohort with 18.0% (*n* = 73) of the patients harboring mCCM is comparable to other published data where the presence of multifocal lesions was described in 10–31% of all cases (20). During 500.5 follow-up years in total, routine follow-up MRI in asymptomatic patients led to an indication for surgery in only 2/73 patients (2.7%) with mCCM. Furthermore, these two cases may need to be critically discussed if surgical intervention was really necessary at all—a spontaneous decrease could have been awaited first. In a 25-year-old patient, without any clinical symptoms, a CCM measuring 2.3 × 2.3 cm in left frontal gyrus was resected due to increase in size during follow-up. Maybe a spontaneous decrease could have been awaited first. In a 23-year-old patient with right-sided hemiparesis due to bleeding of a left-sided ventral CCM of the pons and resection in 2007, surgical resection of a CCM in right anterior putamen was performed due to increase in size. A psychological aspect was considered in this context since the patient had already experienced CCM bleeding and was anxious about new focal neurological deficits due to a new CCM bleeding. Of note, in the non-operated group with 43 patients, 14 patients (32.6%) showed a notable increase in size of at least one CCM lesion during follow-up, but none of them experienced CCM bleeding with focal neurological deficits.

During follow-up, the percentage of patients presenting at the emergency unit due to CCM bleeding with new focal neurological deficits stayed high with 86.4%; in three instances [13.6%; *n* = 3 due to (re-)bleeding of pontine CCM] the patients were referred to our department for second-opinion on surgical treatment options during follow-up. No patient with CCM (re-)bleeding and new focal neurological deficit was seen during routinely performed follow-up.

During the last decade, there has been a controversy whether surgical resection in patients with mCCM and intractable epilepsy should be performed. The presence of mCCM has been shown as predicator of seizure persistence following surgery (15). In our study 4 patients with mCCM underwent surgery due to intractable epilepsy- none of the patients stayed seizure free after surgery.

Considering our data, where routinely performed cMRI had a consequence in only 2/73 (2.7%) asymptomatic patients during 500.5 follow-up years in total, routinely performed follow-up MRI in asymptomatic patients with mCCM is highly questionable as there is no evidence for therapeutic relevance. Furthermore, in case of CCM (re-)bleeding with new focal neurological deficits the patient will most likely present to the emergency unit. Given the fact, that so far no grading system exists for patients with CCM, indication for surgical intervention will only very rarely be performed during consultation in asymptomatic patients. A model based on patients and radiographic factors, which predicts the lifetime rupture risk of CCM in each individual case in order to decide whether surgical treatment should be recommended or not is urgently needed—and could thus dramatically increase the therapeutic relevance of routinely performed follow-up MRI on the one hand and decrease the morbidity due to CCM hemorrhage on the other hand.

In the end, a psychological aspect has to be taken into consideration and patient education plays a major role. Patients who have been actively followed with routinely performed MRI during the last years, might want to proceed with active follow-up based on the idea to achieve certainty over the growth and hemorrhage habit of their CCM lesions. One has to point out to the patients, that no certainty over the growth and hemorrhage habit of their CCM is given and that multiple MRI during lifetime might have an adverse effect on his/her health—as recent findings raise concerns about the context in which gadolinium deposits in the brain (21).

Limitations of our study are: (I) the well-known methodological limitations of retrospective and single-center cohorts (II) relatively small and heterogenous cohort with mCCM (*n* = 73 patients) (III) a potential selection bias since patients with asymptomatic lesions (i.e., CCM hemorrhage without neurological deficits) or a poor preoperative functional status might not have been brought to our attention (IV) the small size of our patient cohort does not offer the opportunity to propose and establish clinical and radiological risk factors for CCM hemorrhage in patients with mCCM (V) despite the topic of follow-up management being of likewise high relevance to patients with single CCM, this is a distinct group of patients and—as such—their management can only be compared to a certain extent to the management of patients harboring multiple CCM and no recommendations are made within this manuscript. Taslimi et al. point out in their meta-analysis, the increased risk of hemorrhage in brainstem CM, as well as rehemorrhage being significantly higher within the first two years after hemorrhage in patients with CCM (22). Horne et al. propose based on their meta-analysis a risk stratification of patients with CCM into four groups [ICH or FND presentation with brainstem CCM (I) or other CCM location (II), as well as other presentation with brainstem CCM (III) or other CCM location (IV)] to predict the 5-year risk of ICH (7). The above-mentioned meta-analyses have to be considered, when proposing a follow-up scheme for patients with single CCM. Data of our patient cohort with single CCM will be published separately and we will try to establish a grading system that can be used in clinical practice in patients with single CCM.

The following illustrative case serves to demonstrate that follow-up imaging can be futile in patients with mCCM.

### Illustrative case report

**Figure 2:**
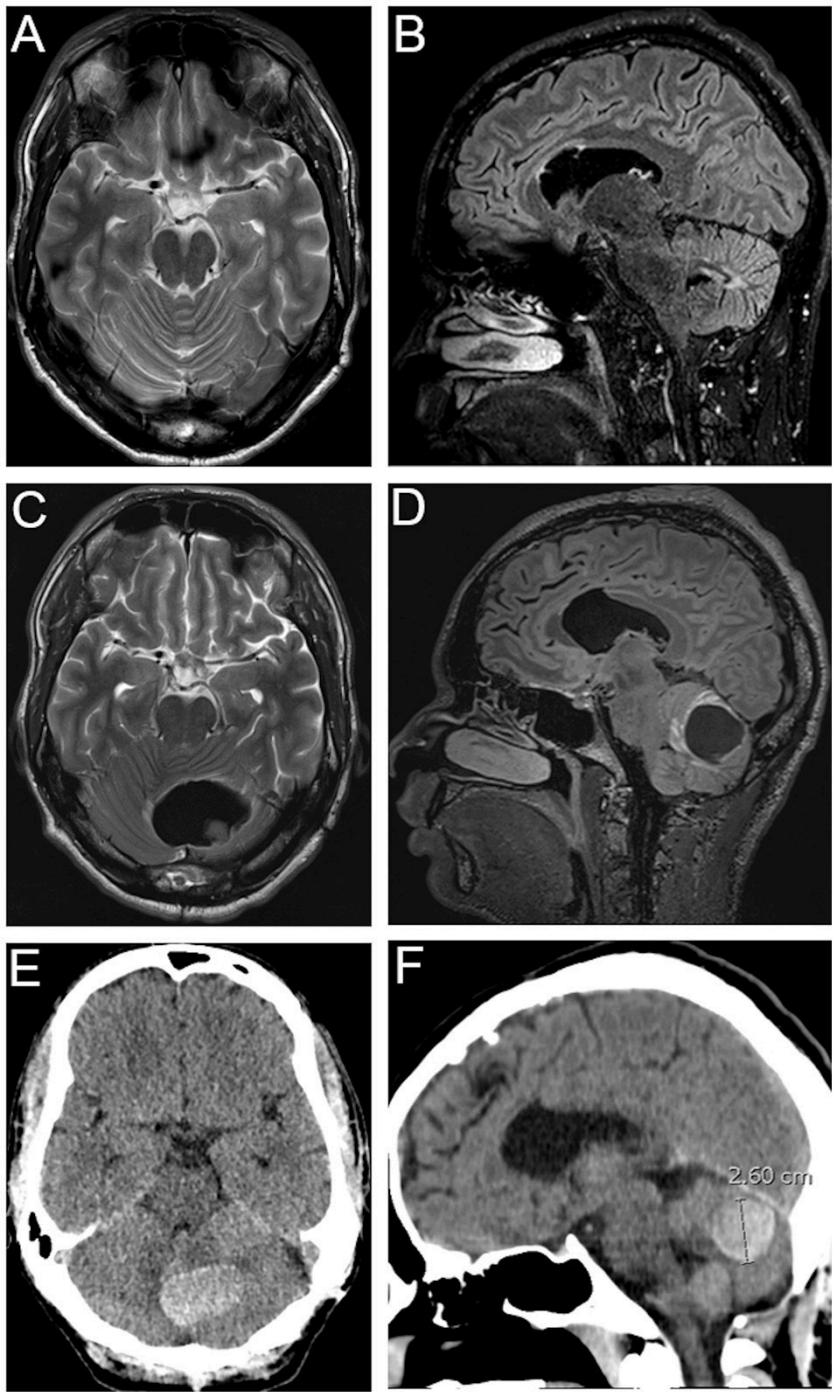
Illustrative case report: Axial **(A)** and Sagittal **(B)** sections of routinely T2-weighted/FLAIR MRI. Axial **(C)** and Sagittal **(D)** sections of T2-weighted/FLAIR MRI and Axial **(E)** and Sagittal **(F)** cCT showing cerebellar CCM bleeding–routinely performed MRI **(A,B)** was performed 7 weeks previously.

Fifteen-year old boy with a cerebellar CCM bleeding and diagnosis of multiple infra- and supratentorial CCM at the age of 15.2. At the age of 25 years the patient presented himself at the emergency unit at our Neurosurgical Department with hemiparetic symptoms on the right-side due to CCM bleeding at the left caudate nucleus. Surgical resection was indicated and performed. In addition, the patient underwent routine cMRI controls every half a year. Nevertheless, the patient presented to our emergency unit five times in total due to CCM bleeding with new focal neurological deficits. Up to date, he underwent an additional resection of a CCM on the left cerebellar hemisphere, a resection of a lesion in the left temporal side and for a second time a CCM resection in the left cerebellar hemisphere. During the last ten years, 20 cerebral imaging were performed—nevertheless routinely performed cerebral imaging had no impact on prevention of CCM bleeding. The shortest duration between routinely performed follow-up MRI showing stable conditions and severe CCM bleeding with acute neurological deficits was just seven weeks (Figure 2).

## Conclusion

Active follow-up by serial imaging is highly questionable and will most likely not have any impact on clinical decision making. Thus, patient education and imaging studies in the setting of new clinical symptoms may be superior to a rigid follow-up imaging schedule in patients with mCCM.

## Ethics statement

This study was carried out in accordance with the recommendations of the University of Zurich. The protocol was approved by the Cantonal Ethics Committee (KEK-ZH; application number 2017-00330). All subjects gave written informed consent in accordance with the Declaration of Helsinki.

## Author contributions

JV conception and design, acquisition of data, analysis and interpretation of data, and drafting the article. MS conception and design, analysis and interpretation of data, and drafting the article. MN conception and design, analysis and interpretation of data, and critically revising the article. YY and LR analysis and interpretation of data and critically revising the article. OB conception and design, acquisition of data, analysis and interpretation of data, drafting the article, and study supervision.

### Conflict of interest statement

The authors declare that the research was conducted in the absence of any commercial or financial relationships that could be construed as a potential conflict of interest.

## Abbreviations

CCM: cerebral cavernous malformations
MRI: magnetic resonance imaging
cMR: cerebral magnetic resonance imaging
FCCM: familial cerebral cavernous malformations
ICH: intracerebral hemorrhage
SWI: susceptibility-weighted imaging
mCCM: multiple cerebral cavernous malformations.
